# Prospecting during egg laying informs incubation recess movements of eastern wild turkeys

**DOI:** 10.1186/s40462-024-00451-3

**Published:** 2024-01-17

**Authors:** Nicholas W. Bakner, Erin E. Ulrey, Bret A. Collier, Michael J. Chamberlain

**Affiliations:** 1 Warnell School of Forestry and Natural Resources, University of Georgia, Athens, GA 30602 USA; 2 School of Renewable Natural Resources, Louisiana State University Agricultural Center, Baton Rouge, LA 70803 USA

**Keywords:** Avian, Behavior, Habitat selection, Prospecting, Recursive movement, Wild turkey

## Abstract

**Background:**

Central place foragers must acquire resources and return to a central location after foraging bouts. During the egg laying (hereafter laying) period, females are constrained to a nest location, thus they must familiarize themselves with resources available within their incubation ranges after nest site selection. Use of prospecting behaviors by individuals to obtain knowledge and identify profitable (e.g., resource rich) locations on the landscape can impact demographic outcomes. As such, prospecting has been used to evaluate nest site quality both before and during the reproductive period for a variety of species.

**Methods:**

Using GPS data collected from female eastern wild turkeys (*Meleagris gallopavo silvestris*) across the southeastern United States, we evaluated if prospecting behaviors were occurring during laying and what landcover factors influenced prospecting. Specifically, we quantified areas prospected during the laying period using a cluster analysis and the return frequency (e.g., recess movements) to clustered laying patches (150-m diameter buffer around a clustered laying period location) during the incubation period.

**Results:**

The average proportion of recess movements to prospected locations was 56.9%. Nest fate was positively influenced (μ of posterior distribution with 95% credible 0.19, 0.06–0.37, probability of direction = 99.8%) by the number of patches (90-m diameter buffer around a clustered laying period location) a female visited during incubation recesses. Females selected for areas closer to the nest site, secondary roads, hardwood forest, mixed pine-hardwood forest, water, and shrub/scrub, whereas they avoided pine forest and open-treeless areas.

**Conclusions:**

Our findings suggest that having a diverse suite of clustered laying patches to support incubation recesses is impactful to nest fate. As such, local conditions within prospected locations during incubation may be key to successful reproductive output by wild turkeys. We suggest that prospecting could be important to other phenological periods. Furthermore, future research should evaluate how prospecting for brood-rearing locations may occur before or during the incubation period.

**Supplementary Information:**

The online version contains supplementary material available at 10.1186/s40462-024-00451-3.

## Introduction

Central place foragers travel from a central location on foraging excursions and return to that location between foraging bouts [53, 70]. Foraging bouts from centralized locations are known to incur a cost of time, energy, and mortality risk [87]. During incubation, avian species are constrained to nest sites, and their ability to use space is restricted if low-risk loafing and foraging areas are not adequately distributed within their range [39, 43, 80]. Therefore, individuals should familiarize themselves with profitable areas within their incubation ranges that provide reduced risk or energetic benefit [52].

Site prospecting is an exploratory behavior common across taxa which allows animals to determine quality of areas within their ranges that would increase fitness [60, 65, 90]. Prospecting occurs at various time periods (pre-, post-, and during reproduction) during the reproductive season [12, 25, 68]. Among avian species, prospecting behavior has been related to identifying migratory stopover [14, 49] and pre- and post-breeding sites [54, 55, 61, 68]. Gathering information using prospecting behaviors can reduce predation risk, increase foraging efficiency, both contributing to individual fitness and reproductive success [60, 65].

Recursive movements are patterns of returns to previously visited areas which occur when individuals identify resources within a heterogeneous landscape [7, 9, 51, 66]. Recursive movement behaviors benefit fitness by improving forage efficiency [64, 81], increasing predator avoidance [79, 86], or in maintaining territories [36, 41]. For central place foragers, prospecting could be used as the mechanism to identify high-quality foraging areas [56], preceding recursive movement to those profitable areas that were identified on the landscape [7].

The onset of egg laying (hereafter laying) and incubation in avian species is an energetically costly time period during which individuals are spatially constrained [22]. Uniparental incubators are faced with the tradeoff of remaining at the nest site or making recess movements (i.e. directional movements made away from nesting location) to gain resources [74, 83]. Prior to incubation, prospecting by females to familiarize themselves with resource distribution could facilitate efficient travel to and from resources while reducing mortality risk [59, 65, 82]. Therefore, prospecting during the laying period could be important in supporting behavioral strategies used during incubation [59, 65].

Female eastern wild turkeys (*Meleagris gallopavo silvestris*; hereafter wild turkey) are uniparental ground nesters that maintain ranges, but do not defend territories [32]. During nesting, females are central-place foragers that make foraging bouts from the nest location during incubation [3, 18, 45]. To survive the incubation period, females identify resources that provide foraging opportunities and concealment from predators (Green 1982, [3, 45, 85]. Contemporary research has shown that prior to laying, female wild turkeys do not prospect for potential nest sites [17], but it is plausible that individuals may prospect for resources during the laying period [16]. Furthermore, pre-nesting and laying ranges show little overlap [71], and during laying, females increase daily movements but decrease space use, indicative of a lack of site familiarity [31, 48, 71]. It is plausible that movement behaviors during the laying period may maximize foraging success and reduce predation risk during the incubation period [2, 11]. However, it is unclear if areas identified by females during laying are ultimately selected and visited during incubation when females take incubation recesses [3, 45].

Our objectives were to (1) determine if female wild turkeys returned to locations prospected during the laying period and how many patches were used when making recess movements during incubation, and to (2) assess the relationship between environmental and movement covariates during incubation recesses to areas they prospected during the laying period. Prospecting behavior in avian species is known to enhance foraging ability and reduce predation risk during reproduction by increasing landscape familiarity (Pärt and Doligez 2011). During incubation, avian species must balance incubating the nest and ensuring their own survival, making the identification of resources crucial for maintaining their survival during this period [22]. Notably, recess movements during the incubation period significantly influence wild turkey nest success [3, 45]. Therefore, we hypothesized that incubating females would return to sites previously visited during the laying period when taking incubation recesses, and such behaviors would positively affect nest fate. Specifically, we predicted that females who did not revisit sites previously visited during laying would have lower nest success. If incubation recesses occurred in locations prospected during laying, it could indicate the presence of high-quality forage or closer proximity to the nest, potentially reducing overall movements [3, 45]. Therefore, we hypothesized sites prospected during laying and selected by incubating females would be closer to landcover that provide foraging opportunity but reduce their distance from the nest location. Specifically, we predicted that females would select for pine and open landcover types that are closer to the nest. Implications from our findings offer a novel perspective on the timing of prospecting behavior and its impact on nest fate, providing contributions to the understanding of spatial–temporal resource selection.

## Methods

We used rocket nets to capture wild turkeys from January-March of 2014–2021 (For details on study sites refer to Additional file 1). We aged captured individuals based on presence of barring on the ninth and tenth primary feathers and sexed them by the coloration of the breast feathers [58]. We banded each bird with an aluminum rivet leg band (National Band and Tag Company, Newport, Kentucky,female size = 8, male size = 9) and radio-tagged each individual with a backpack-style GPS-VHF transmitter [30] produced by Biotrack Ltd. (Wareham, Dorset, UK). We programmed transmitters to record 1 GPS location nightly (23:58:58) and hourly GPS locations from 0500 to 2000 (Standard Time and according to the appropriate time zones) for the duration of the study [15]. Each transmitter had a mortality switch that was programmed to activate after > 23 h of no movement. We released turkeys immediately at the capture location after processing. All turkey capture, handling, and marking procedures were approved by the Institutional Animal Care and Use Committee at the University of Georgia (Protocol #A2019 01-025-R2 and #A2020 06-018-R1) and the Louisiana State University Agricultural Center (Protocol #A2014-013, A2015-07, and A2018-13).

We located wild turkeys ≥ 2 times per week using a 3-element handheld Yagi antenna and receiver to monitor survival based on the presence of a mortality signal, general movements of individuals within their ranges, and onset of nesting activity. We remotely downloaded GPS locations from each turkey ≥ 1 time per week. In ArcGIS 10.8 (Environment Systems Research Institute, Redlands, California, USA), we spatially projected GPS locations to identify nest locations by determining when a female’s locations became concentrated, which represented the onset of incubation [3, 18]. When VHF tracking and GPS locations indicated nest termination, we located the nest site to determine if hatching had occurred [17, 88, 89].

We processed and cleaned the raw GPS data by removing fix locations that had dilution of precision values (DOP) > 7 which is considered error in the positional fix due to the geometry of the satellite signal received [29]. To determine dates of nest initiation (i.e. initiation of laying) and onset of incubation initiation, we mapped our spatial–temporal data using ArcGIS 10.8 (Environment Systems Research Institute, Redlands, California, USA). We identified the onset of incubation as the first time an individual remained on the nest overnight [3, 45], and then evaluated hourly locations for the previous 20 days to determine when a female initially visited the nest site (defined as location being < 20 m from the known nest site, [17, 18, 71]. We considered the date of first visit as the date of nest initiation and used it as the beginning of the laying period as wild turkeys rarely visit nest sites before laying the first egg [16, 17]. Incubation recesses are directional movements made away from nesting locations during active incubation, which are thought to allow individuals time to acquire necessary resources while maintaining appropriate egg temperatures [22]. Following Bakner et al. [3], we classified recess movements during incubation as any location > 27.5 m (27.5 m is associated with the 90th percentile error of the transmitter) away from the known nest location and all other locations (< 27.5 m) as incubation and not recessing. We calculated the distance each recess location (any point > 27.5 m away from the nest) was from the nest location (distance to nest) to incorporate into our model to evaluate habitat selection. We performed data processing and analysis in program R (v.4.1.0; [63].

### Covariates

**Fig. 1:**
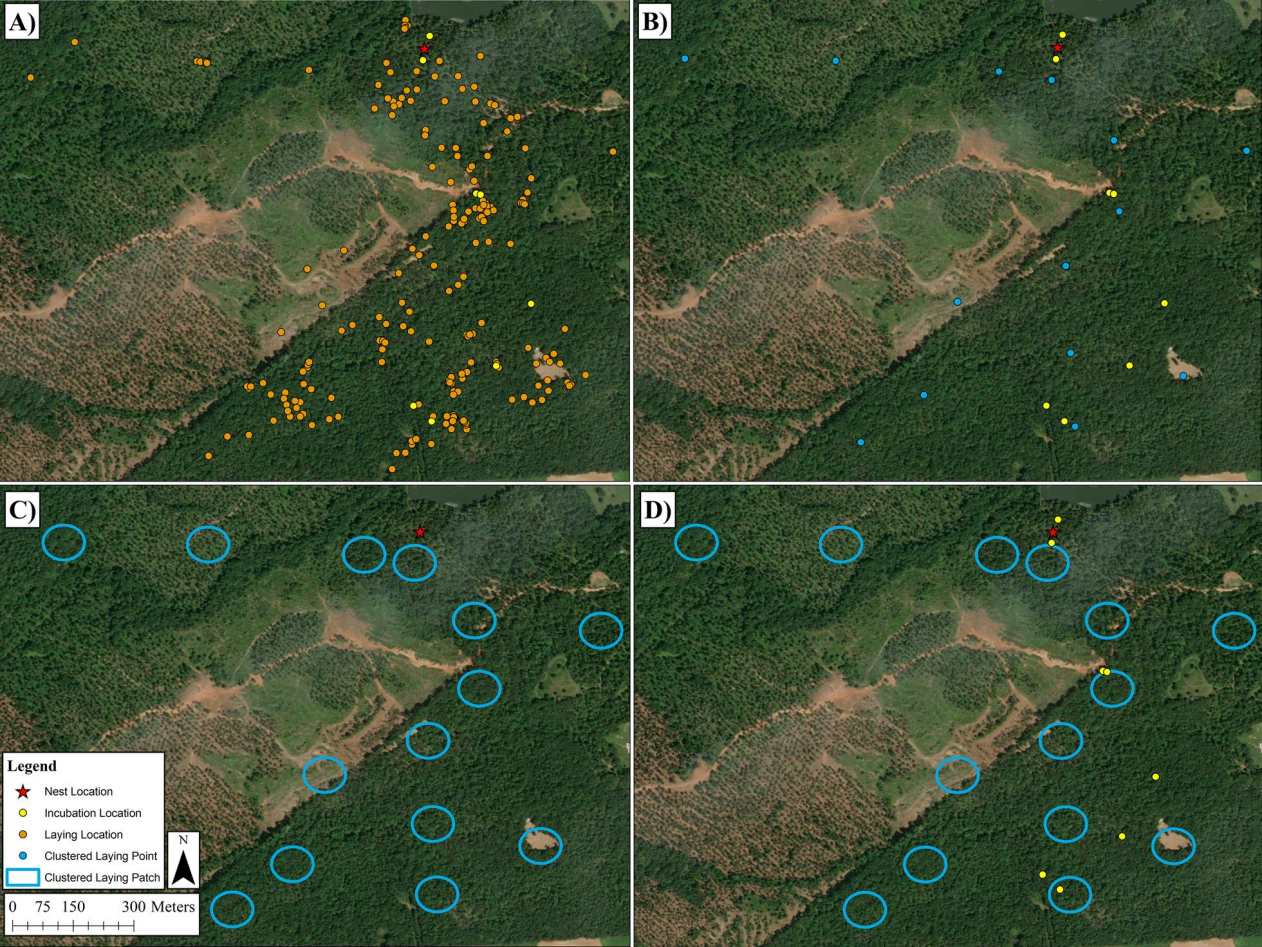
GPS locations of a female eastern wild turkey depicting how we determined the CLP covariate and number of incubation recesses. **A** Laying and incubation movements used for the analysis with a star showing the nest location. **B** Clustered laying period GPS locations created from the cluster analysis (150 m radius buffer; hereafter, CLP). **C** The CLP with a 45 m radius buffer determined from how far a female travels during incubation. **D** Any incubation point that fell within a CLP contributed to the proportion of recesses made to a laying period location. Any CLP that contained an incubation location was considered a CLP that was used

We assigned a unique identification to each female GPS location for the duration of the laying period. To quantify location-specific revisitation for individual females, we combined laying locations that were to the same areas using a cluster analysis in package geosphere in program R [33], v.4.1.0; [63]. Using estimates from Schofield [71] who reported that female wild turkeys moved 300 m/hr during the laying period, we used a 150-m radius buffer of each unique GPS location to perform the cluster analysis (Fig. 1). Specifically, we used a 150-m radius buffer to account for average movement during the laying period, preventing the double-counting of laying period locations and treating multiple locations within the same area as one. The cluster analysis allowed us to associate each female with a set of potential prospecting locations. We then used the clustered laying period locations to quantify how many incubation recesses were made to that area.

Following Bracis et al. [8], we calculated the revisit rate to evaluate whether incubation recess movements by females were to locations previously visited during the laying period, we used package recurse in R (v.4.1.0,[63]. We first assigned a unique identification to each of the clustered laying patch (hereafter, CLP. We used estimates of daily distance traveled while on an incubation recess from Bakner et al. [3], 90 m) to set an appropriate circular buffer size around each CLP. We then used the function getRecursionsAtLocation in package recurse in R [8],v.4.1.0; [63] to calculate how many incubation recesses locations fell within a 90 m diameter circular buffer of a CLP. Specifically, the function getRecursionsAtLocation allowed us to evaluate how many times an incubation recess movement was to a CLP.

First we quantified the proportion of recess movements that went to a CLP. We calculated the proportion of recess movements made to CLP by using the number of recesses to any CLP and dividing by the total number of recesses during the incubation period. We then counted the total number of CLP used during the incubation period. Specifically, to calculate the number of CLP used during incubation, we counted the number of CLP that were visited at least once during incubation recess.

Female wild turkeys are constrained to the nest location during incubation [3, 45]. Thus, we calculated the distance from the nest location to the recess movement locations to see if this distance influenced habitat selection. Understanding patterns of resource selection relative to the presence of recursive movements offers a mechanism to link resource availability and female behavioral decisions [2]. So, we evaluated resource selection using a set of landcover covariates relevant to female wild turkey reproductive ecology [3, 13, 45]. We obtained year-specific, 30-m resolution spatial data on landcover from the Cropland Data Layer (Cropscape provided by the National Agricultural Statistics Service (National Agricultural Statistics Service 2015. We recoded and combined landcover in program R (v.4.1.0 [63], to create 6 unique landcover types (water, pine forest, hardwood forest, mixed pine-hardwood forest, open treeless areas, and shrub/scrub, [88, 89]). We calculated the nearest distance from each turkey use and available points to each landcover type, using the Euclidean distance tool in ArcMap 10.8 (Esri, Redlands, CA, USA). We used landcover distance metrics for subsequent analysis instead of a classification or categorical approach [19].

### Nest fate model

We constructed a Bayesian logistic regression model to test our hypothesis regarding the relative importance of incubating females revisiting sites previously visited during laying on nest fate. Specifically, we included the covariates proportion of recess movements to CLP and number of CLP visited to predict nest fate. We chose the proportion of recess movements to CLP as a predictor because it reflects the proportion of movements back to prospected locations. Additionally, the choice of incorporating the number of CLP as a predictor was due to the potential benefits associated with having a greater variety of places to recess. We treated the probability of nest fate (success or failure) as a Bernoulli distribution. Our model included a unique identification number for each female turkey as a random effect to account for inter-individual variation. To improve model fit and allow for direct comparison of effect sizes of each predictor variable, we normalized all fixed effects included in the models using the scale function in R. We fitted models using package brms in program R [10]. We computed 4 MCMC chains for 8000 iterations, discarding the first 1000 iterations as a burn-in [28]. We calculated 95% credible intervals that provided a metric of uncertainty. We then computed the probability of direction which provided the probability each covariate either positively or negatively influenced nest fate. All estimated parameters had R-hat values < 1.1, indicating that all chains converged [26].

### Resource selection model

We calculated 95% home ranges during the incubation period by fitting dynamic Brownian bridge movement models (dBBMMs) to the time-specific location data [15] using package move [42] in program R. We used an error estimate of 20 m, a moving window size of 7 locations, and a margin setting of 3 locations [11, 15]. These home ranges were estimated to evaluate resource selection within an individual’s incubation range.

We used resource selection functions (RSFs) to examine relationships between 6 landcover types and distances traveled from nests to wild turkey incubation recess movements to CLP within individual incubation ranges (3rd-order selection) following design III approach suggested by Manly et al. [46]. We compared use (incubation recess movements to CLP) points within individual incubation ranges to 500 available points sampled within each range [6]. We tested for collinearity between each of our covariates and excluded covariates using Pearson’s correlation with a *r* > 0.60 [24]. We found no correlation among covariates in our model. We used a generalized linear mixed model to include a random intercept for each individual turkey, with a binomial response distribution and logit link to the used-available data for turkeys [38, 46]. We used the lme4 R package [4] with a binary (0 = available, 1 = used) response variable to model resource selection. To improve fit, we rescaled all fixed effects by subtracting their mean and dividing by 2 standard deviations prior to modeling [27]. Due to the exploratory nature of our study, we chose to not use a model selection methodology but, instead, used a global model using the covariates selected for their known importance in wild turkey ecology.

## Results

**Fig. 2:**
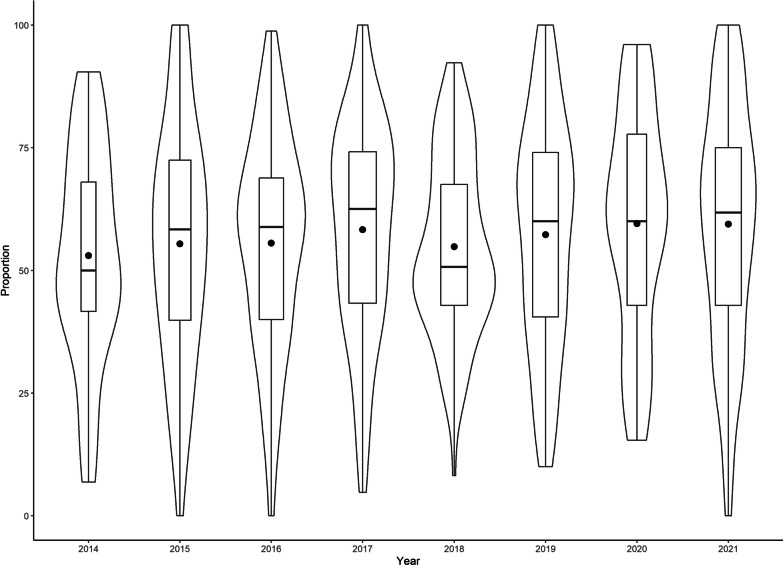
Proportion of incubation recess movements made to CLP for 585 nesting attempts made by 435 female eastern wild turkeys (*Meleagris gallopavo silvestris*) across the southeastern United States during 2014–2021. Violin plots are the distribution of the data with corresponding boxplot inside. The solid line identifies the median and the dot corresponds with the average proportion

**Fig. 3:**
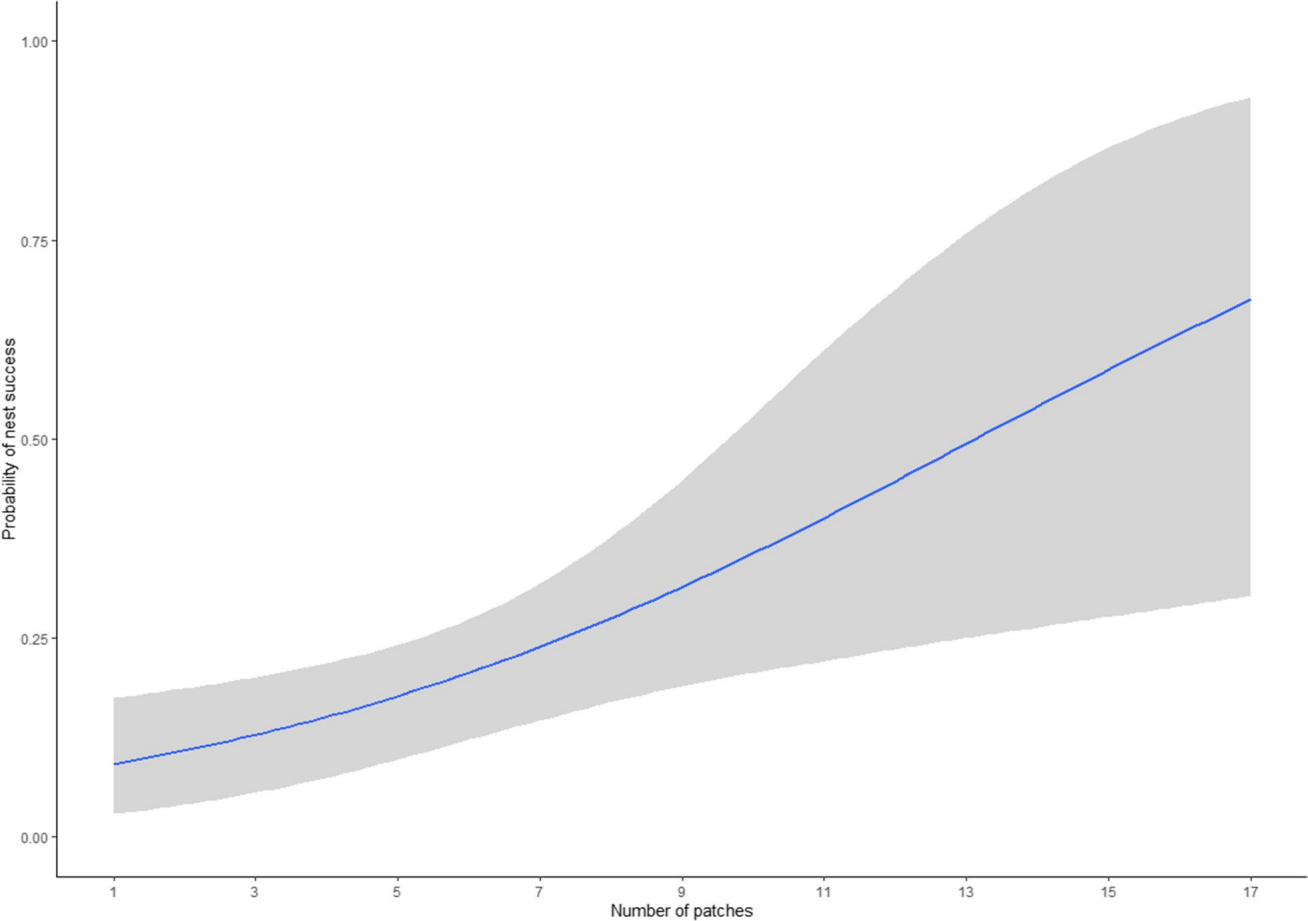
Probability of nest success as a function of the number of CLP visited during incubation recesses for 585 nesting attempts made by 435 female eastern wild turkeys (*Meleagris gallopavo silvestris*) across the southeastern United States during 2014–2021. Gray shading represents the 95% credible intervals

We monitored 692 nesting attempts by 485 (427 adults and 55 juveniles, 3 unknown) female wild turkeys during 2014‒2021. We removed 107 nesting attempts that were incubated < 3 days since we were unable to isolate incubation behaviors from nests of such short duration. We used 585 nesting attempts (initial attempts = 407, renesting attempts = 178) by 435 females to quantify whether females were revisiting CLP. We identified 31,145 recess movements during incubation, of which 56.9% (SD = 22.2, median = 58.7) were made to CLP (Fig. 2). Mean number of CLP used during laying that were visited during incubation recesses was 5 (SD = 1.9, range = 0–17 patches). The random effect of individual had a variance of 1.44 ± 0.63 within our nest fate model. The proportion of recess movements to CLP had no effect on nest fate (μ of posterior distribution with 95% credible  − 0.0,  − 0.01 to 0.01, probability of direction = 61.6%). However, as the number of CLP used during incubation recesses increased there was a positive impact on nest fate (μ of posterior distribution with 95% credible 0.19, 0.06–0.37, probability of direction = 99.8%), where the probability of nest success increased by 2.8% for every additional CLP visited (Fig. 3).

**Fig. 4:**
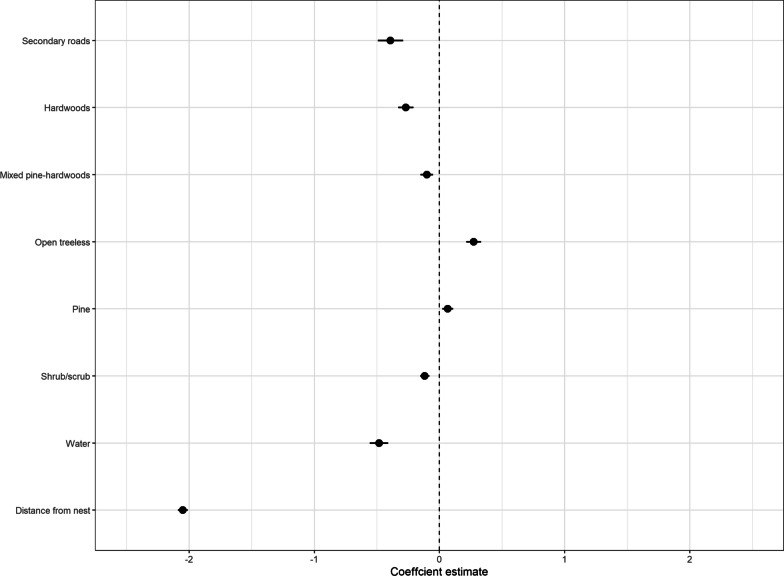
Coefficient plot depicting resource selection of eastern wild turkeys (*Meleagris gallopavo silvestris*) during incubation recesses to CLP across the southeastern United States during 2014–2021. The whiskers depict 95% confidence intervals around regression coefficient estimates

For our RSF, we used 16,278 GPS locations from recess locations that were to 2831 CLP and created 585 individual home ranges. The random effect of individual had a variance of 1.84 (SE ± 1.36) in our RSF. Female wild turkeys selected for areas closer to hardwoods (β =  − 0.27, SE ± 0.031), water (β =  − 0.48, SE ± 0.037), mixed pine-hardwoods (β =  − 0.10, SE ± 0.025), secondary roads (β =  − 0.39, SE ± 0.051), shrub/scrub (β =  − 0.16, SE ± 0.583), and areas closer to the nest (β =  − 2.04, SE = 0.020; Fig. 4). Female wild turkeys avoided areas closer to open treeless areas (β = 0.27, SE ± 0.030) and pine (β = 0.06, SE ± 0.022; Fig. 4).

## Discussion

Prospecting behavior before the onset of incubation has been found to occur in a variety of avian species [65]. Presumably, species rely on prospecting to determine areas capable of conferring greater nest success [23] and profitable patches ensuring availability of resources [56]. Using prospecting movements, our results indicate that ~ 57% of incubation recess movements were to patches visited during laying. Our findings support contemporary research demonstrating that wild turkeys increase daily movements during laying, indicative of a lack of site familiarity [71]. Similar behaviors have been described in waterfowl (*Anas* sp.) that visit future brood-rearing ponds prior to hatching [12], ruff (*Philomachus pugnax*) and black grouse (*Tetrao tetrix*) where females visit leks prior to the breeding season [5] and is presumed to occur in sage grouse (*Centrocercus urophasianus*) prior to incubation [25].

We observed that nest fate was not influenced by the number of times a female returned to CLP but was affected by how many different CLP she visited while on incubation recesses. Observational work by Williams and Austin [84] reported the unpredictability of timing and movement patterns by female wild turkeys during incubation. When individuals are faced with patchy resource distributions, they become constrained by the spatial distribution of resources [72]. Where resources are sparse, prey may have to endure periods of overlap with predators which makes prey more predictable, providing cues into their nesting behavior [69, 72]. Alternatively, when prey are surrounded by multiple safe sites where predators are less efficient, predators may avoid these locations [73]. Having multiple profitable foraging patches allows prey to be more unpredictable in their movements which favors the prey’s behaviors during nesting instead of the predator [75], thus, reducing cues to nest site locations [37]. Moreover, the familiarity of sites due to prospecting behavior could play a crucial role in reducing predation risks. Site familiarity may lead to a decrease in the duration and number of incubation recess bouts, a factor known to influence predation in many ground-nesting bird species [21, 44, 45, 76]. Our findings suggest that female wild turkeys not confined to repeatedly using the same patches within their incubation ranges had increased nest success. Collectively, these findings highlight the importance of site familiarity in shaping nesting behaviors and ultimately influencing nest success in the context of predator–prey dynamics. Overall, we found that female wild turkeys that were not confined to repeatedly using the same patches within their incubation ranges had increased nest success.

During nesting, avian species should surround themselves with adequate resources to survive incubation while reducing predation risk [22, 74]. Wild turkeys are habitat generalists [62], so we were not surprised that females used a variety of landcover types during incubation recess movements to sites previously visited during laying. Presumably, females were simply going to places that offered conditions capable of supporting survival. Our results indicated that pine and open-treeless areas were avoided by wild turkeys. Open-treeless areas and pine forest on our sites were typically open pastures dominated by forages planted for livestock, sod-forming grasses, or industrial pine forest. Similar types of open areas and pine forest fail to offer high quality foraging habitats for incubating females relative to other early successional vegetation communities [1, 47]. Likewise, during incubation females often try to avoid other females, hence reducing predation risk [32, 67, 71]. Therefore, remaining in forested areas could provide concealment to reduce intraspecific interactions and predation risk. Alternatively, environmental thermal regimes can shape avian behavior [35], and in warmer environments, Galliformes have been found to adjust habitat use to select for areas with cooler temperatures [34, 77]. Specific to wild turkeys, Nelson et al. [50] found that broods on our study sites avoided pine forests and selected cooler locations as the day progressed. Therefore, avoidance of open-treeless areas and pine forest may be due to thermal regulatory constraints.

Our results emphasize the complexity of how prior behavioral processes can affect future events, such as nesting behavior. While researchers often concentrate on characterizing nest sites, there is a tendency to neglect the critical aspect of movement decisions [22]. This neglects results in characteristics that frequently fail to describe the spatial scale at which nesting occurs [22, 78] and are not clearly linked to nest success [20, 40]. Our research highlights the biologically relevant spatial and temporal scale at which prospecting decisions influence nest fate. We also provide a different approach to understanding resource selection during avian reproduction. Specifically, we suggest that focus on identifying resource selection and activities during the laying period could be relevant and appropriate for other ground-nesting species. Furthermore, prospecting behavior has been thought to occur during recess bouts to identify brood-rearing habitat [25]. Future research should evaluate how prospecting for brood-rearing habitat may occur prior to or during the incubation period.

## Conclusions

Central place foragers are faced with understanding their surroundings to maximize foraging ability while reducing predation risk. Prospecting behavior allows individuals to determine suitability of areas within their ranges that would increase fitness. We found that wild turkeys frequently (~ 57%) return to locations previously visited during the laying period. Furthermore, we found that this behavior was common among wild turkeys, but nest fate was influenced by numbers of prospected locations within the incubation range. Our findings suggest that having more patches could reduce cues to predators and positively influence nest fate. Alternatively, increased site familiarity through prospecting could reduce incubation recess bouts and duration which positively influence nest fate.

### Supplementary Information

**Additional file 1: **Study site descriptions.

## Data Availability

The dataset supporting the conclusions of this article is archived in the Dryad repository.

## Abbreviations

CLP: Clustered laying patch
GPS: Global positioning system
RSFs: Resources selection function
VHF: Very high frequency
