# Supplementary material for: Prospecting during egg laying informs incubation recess movements of eastern wild turkeys
Source: Mov Ecol. 2024 Jan 17;12:4. doi: 10.1186/s40462-024-00451-3 (PMC10792941; doi:10.1186/s40462-024-00451-3)
Supplement: Supplementary file 1 — Additional file 1: Study site descriptions. [file 40462_2024_451_MOESM1_ESM.docx]

N.W. Bakner^1^ *, E.E. Ulrey^1^, B.A. Collier^2^, and M.J. Chamberlain^1^

^1^Warnell School of Forestry and Natural Resources, University of Georgia, Athens, GA 30602, USA

^2^School of Renewable Natural Resources, Louisiana State University Agricultural Center, Baton Rouge, LA 70803, USA

*Corresponding author; Email:  [Nicholas.Bakner@uga.edu](mailto:%20Nicholas.Bakner@uga.edu)

**Prospecting during egg laying informs incubation recess movements of eastern wild turkeys**

Section S1. Study Area

We conducted research on 11 sites across the southeastern United States. In Louisiana, we conducted research on the Kisatchie National Forest (KNF), Fort Polk Wildlife Management Area (WMA), and Peason Ridge WMA from January 2014-August 2021. The KNF was owned and managed by the United States Forest Service (USFS), whereas Fort Polk and Peason Ridge WMA was jointly owned by the USFS and the United States Army. Louisiana sites were composed of pine (*Pinus* spp.)-dominated forests, hardwood riparian zones, and forested wetlands, with forest openings, utility right-of-ways, and forest roads distributed throughout. Primary overstory species included longleaf pine (*Pinus palustris*), loblolly pine (*P. taeda*), oaks (*Quercus* spp.), hickories (*Carya* spp.), and red maple (*Acer rubrum*). For a detailed description of site conditions on KNF and Fort Polk WMA see Yeldell et al. (2017*a,b*).

We conducted research on Lake Seminole and Silver Lake WMAs located in southwest Georgia from January 2015-August 2016. The Silver Lake WMA was owned and managed by the Georgia Department of Natural Resources-Wildlife Resources Division (GADNR), and the adjacent Lake Seminole WMA was owned by the U.S. Army Corps of Engineers and managed by GADNR. Both sites were predominantly mature pine forests and forested wetlands. Overstory species were predominately longleaf pine, loblolly pine, slash pine (*P. elliottii*), oaks, and sweetgum (*Liquidambar styraciflua*). For a detailed description of site conditions on Silver Lake WMA see Wood et al. (2019).

We conducted research on B.F. Grant and Cedar Creek WMAs located in the Piedmont region of Georgia from January 2017-August 2021. B.F. Grant WMA was owned by the Warnell School of Forestry and Natural Resources at the University of Georgia, and was managed jointly by the GADNR and the Warnell School. B.F. Grant WMA landcover was primarily loblolly pine forest, agricultural lands, mixed hardwood and pine forests, and hardwood lowlands containing mostly oaks, sweet gum, and hickory. Agricultural lands were mostly grazed mixed fescue (*Festuca* sp*.*) fields and hay fields planted for ryegrass (*Lolium* sp*.*). Cedar Creek WMA was owned by the USFS and managed in partnership with GADNR. Cedar Creek WMA was composed primarily of loblolly pine uplands, mixed hardwood and pine forests, and hardwood lowlands of similar species composition as B.F. Grant WMA. For a detailed description of site conditions see Wakefield et al. (2020).

We conducted research on 3 contiguous WMAs (Webb, Hamilton Ridge, and Palachucola; hereafter, Webb WMA Complex; January 2014-August 2018) and the Savanah River Site (hereafter, SRS; January 2021-March 2021) in South Carolina. The Webb WMA Complex was owned and managed by the South Carolina Department of Natural Resources (SCDNR). The Webb WMA Complex was dominated by longleaf, loblolly, and slash pine forests with hardwood stands along riparian corridors, and expanses of bottomland hardwood wetlands. The SRS was owned by the United States Department of Energy and managed by USFS. The SRS was primarily forested and consisted of bottomland hardwoods, mixed-pine hardwoods, and planted stands of longleaf pine, loblolly pine and slash pine. For a detailed description of site conditions see Wightman et al. (2019).

**References**

Wakefield, C.T., Martin, J.A., Wightman, P.H., Bond, B.T., Lowrey, D.K., Cohen, B.S., Collier, B.A. & Chamberlain, M.J. (2020). Hunting activity effects on roost selection by male wild turkeys. *Journal of Wildlife Management,* **84**(3), 458-467.

Wightman, P.H., Kilgo, J.C., Vukovich, M., Cantrell, J.R., Ruth, C.R., Cohen, B.S., Chamberlain, M.J. & Collier, B.A. (2019). Gobbling chronology of eastern wild turkeys in South Carolina. *Journal of Wildlife Management*, **83**(2), 325-333.

Wood, J.D., Cohen, B.S., Conner, L.M., Collier, B.A. & Chamberlain, M.J. (2019). Nest and brood site selection of eastern wild turkeys. *Journal of Wildlife Management,* **83**(1), 192- 204.

Yeldell, N.A., Cohen, B.S., Little, A.R., Collier, B.A. & Chamberlain, M.J. (2017) *a*. Nest site selection and nest survival of eastern wild turkeys in a pyric landscape. *Journal of Wildlife Management,* **81**(6), 1073-1083.

Yeldell, N.A., Cohen, B.S., Prebyl, T.J., Collier, B.A. & Chamberlain, M.J. (2017) *b*. Prescribed fire influences habitat selection of female eastern wild turkeys. *Journal of Wildlife Management,* **81**(7), 1287-1297.
